# Antioxidant system of garden cress sprouts for using in bio-monitor of cadmium and lead contamination

**DOI:** 10.1038/s41598-023-37430-4

**Published:** 2023-06-27

**Authors:** Azza M. Abdel-Aty, Alshaimaa M. Elsayed, Abdul Aziz M. Gad, Amal Z. Barakat, Saleh A. Mohamed

**Affiliations:** Molecular Biology Department, National Research Centre, Dokki, Cairo, Egypt

**Keywords:** Biochemistry, Plant sciences

## Abstract

Based on garden cress significantly used for phytoremediation, the antioxidant system included antioxidant-phenolic compounds and antioxidant-enzymes of 6-day-garden cress sprouts (GCS) were assessed as potential bio-indicators for cadmium (Cd) and lead (Pb) contamination. Total phenolic and flavonoid contents of GCS germinated under Cd and Pb treatments (25–150 mg kg^−1^) gradually increased with increasing concentration of metals and peaked by 2.0, 2.6, and 2.5, 2.3 folds at 150 mg kg^−1^, respectively. By using DPPH, ABTS, and PMC antioxidant assays, the total antioxidant activity of phenolic compounds of GCS increased 6.1, 13.0, and 5.8-fold for Cd and 5.9, 14.6, and 8.2-fold for Pb at 150 mg kg^−1^, respectively. The antioxidant enzymes of GCS (POD, CAT, GR, and GST) were significantly activated in response to Cd and Pb stress, and two new electrophoretic POD bands were detected. GCS was absorbed 19.0% and 21.3% of Cd and Pb at 150 mg metal kg^−1^, respectively. In conclusion, the approaches of the antioxidant defense system of GSC could potentially be used as bio-indicator for monitoring Cd and Pb contamination in a short time of germination process.

## Introduction

Heavy metals are some of the most pervasive and severe environmental pollutants in the world. Among them, cadmium (Cd) and lead (Pb) are the most common and hazardous; both can have harmful effects on human health. Cd is a potent nephron toxin, a class one carcinogen, and is associated with many severe diseases including teratogenic consequences, hypertension, liver damage, lung damage, renal malfunction, urine protein spills, and disruption of protein metabolism. Acute and/or persistent exposure to Pb is also related to substantial harm to the kidney, nervous system, reproductive system, liver, and brain in addition to metabolic toxicity and enzyme inhibitors^1–3^. Cd and Pb, among other heavy metals, flow and leak into the soil–plant system from many sources such as sewage sludge, ash, inorganic fertilizers, atmospheric deposition, and base and precious mining activity, as well as waste byproducts from petrochemical industries^3^. Wastes like ash and sewage sludge contain large quantities of rapidly biodegradable organic materials, subsequently causing the release of hazardous heavy metals into the environment^4^.

Various concentrations of Pb and Cd were measured in different Egyptian soils collected from some regions, ranging between 32.22–88.67 mg Pb kg^−1^ and 11.35–28.88 mg Cd kg^−1^^5^. The average concentration of Pb and Cd in these studied agricultural soils was greater than their maximum allowable concentrations reported by WHO^6^ as 84 and 4 mg Kg^−1^, respectively. These indicate a serious Pb and Cd pollution danger affecting soil quality and requiring more attention. However, El-Hassanin et al.^7^ reported concentrations of Pb and Cd in samples collected from other Egyptian soils ranging from 1.77 to 7.33 mg Pb kg^−1^ and 0.40 to 1.66 mg Cd kg^−1^. Moreover, the Cd pollution in some soils via phosphate fertilizers, atmospheric deposition, and solid waste leaching reach 77, 74, and 344 mg kg^1^ in the soil, respectively^8^.

To address the heavy metal soil contamination problem, several physical and chemical remediation technologies have been developed^9^. However, these remediation technologies are typically expensive, cause damage to the soil, and produce conditions that are unsuitable for plant growth. Phytoremediation is a promising, clean, and cheap technology, receiving attention from soil scientists and the general public. Several phytoremediation approaches have been used for the remediation of heavy metals from polluted soils. Some plant species were used in phyto-stabilization to reduce heavy metal migration in belowground soil, phyto-extraction to absorb heavy metals from containment soil, phyto-volatilization to release heavy metals into the atmosphere, and phyto-filtration to adsorb heavy metals from groundwater^9,10^. Phytoextraction is the most significant phytoremediation method used nowadays, as a permanent solution, for removing heavy metals from contaminated soil. In phytoextraction, the heavy metals were taken up by plant roots, translocated to their aerial parts, and then sequestrated in the different plant tissues^11^. Hyperaccumulator plants can collect great amounts of heavy metals in their aboveground tissues without any signs of phytotoxicity. The heavy metal hyperaccumulator plants can collect 100 times more than ordinary plants under the same conditions. Currently, metal hyperaccumulators include more than 450 plants from at least 45 angiosperm families^12^.

The common signs of heavy metal toxicity are plant growth inhibition, photosynthesis reduction, and disturbances of respiration and nitrogen metabolism^13,14^. In addition, heavy metals cause oxidative stress/damage through the overproduction of reactive oxygen species (ROS), which attack plant cells to cause lipid peroxidation, protein oxidation, nucleic acid damage, and ultimately cell death^15^. To combat such oxidative damage, plants can alter the activities of various antioxidant enzymes, including ascorbate peroxidase (APX), catalase (CAT), glutathione reductase (GR), guaiacol peroxidase (POD), glutathione S-transferase (GST), and overproduce antioxidant phenolic compounds^16^. These changes in antioxidant enzymes and nonenzymatic antioxidant phenolic compounds are considered biomarkers for monitoring soil heavy metal contamination^13,17^.

Garden cress (*Lepidium sativum* L.) is a fast-growing edible plant that belongs to the Brassicaceae family. It grows in many countries, is native to Egypt, and is used for several culinary and medicinal purposes. It can be grown in any type of climate under various soil conditions and can be sown and harvested several times during the year^18^. In addition, its short vegetation period and high sensitivity to slight changes in environmental conditions make it suitable for environmental stress investigations^19^. Several studies reported that the full garden cress plant can be used for phytoextraction of residual soil ecotoxicity and the edible parts of the plant accumulated relatively high concentrations of heavy metals^19–22^. Some of the above studies used the enhancement of antioxidant-phenolic compounds and antioxidant enzymes as bio-indicators for plants cultivated in metal-polluted soil, but the plants take a long time to cultivate. However, our recent study showed that 6-day- garden cress sprouts possessed the highest content of antioxidant-phenolic compounds along with the highest activities of antioxidant enzymes^23^. Therefore, this study was conducted to assess the garden cress sprouts (GCS) antioxidant defense system, including the total phenolic content, total flavonoid content, and total antioxidant activity of their phenolic compounds, and variation in their antioxidant enzyme activities (POD, CAT, GR, and GST) as potential bio-indicators for monitoring the cadmium (Cd) and lead (Pb) contamination at various concentrations after 6 days of germination (a short period of germination). This study will provide important information on the potential of garden cress six-day sprouts as Cd/Pb indicators within a very short time.

## Materials and methods

### Chemical reagents

Folin Ciocalteu reagent, ammonium molybdate, 1,1-diphenyl-2-picrylhydrazyl (DPPH), 2,2-azinobis (3-ethylbenzo-thiazoline-6-sulfonic acid) (ABTS), Guaiacol, H_2_O_2_, 1-Chloro-2,4-dinitrobenzene (CDNB), nicotinamide adenine dinucleotide phosphate (NADPH), oxidized glutathione (GSSG) were purchased from Sigma-Aldrich Co.

#### Seed source

The seeds of *L. sativum* garden cress plants were obtained from the Agriculture Research Center, Cairo, Egypt. The formal identification of the plant is confirmed in a voucher sample (Ser. No. 5220) deposited at Herbarium, Agriculture Research Center. Experimental research and field studies on the *L. sativum*-garden cress plant (either cultivated or wild), including the collection of plant material, complied with relevant institutional, national, and international guidelines and legislation.

#### Germination of garden cress seeds in contaminated media

Germination of garden cress seeds was carried out according to Abdel-Aty et al.^23^. Garden cress seeds were surface-sterilized with 0.1% (v/v) sodium hypochlorite solution after which they were extensively washed with distilled water. CdCl_2_ and PbCl_2_ were individually dissolved in distilled water and mixed with cotton as a growth medium at different concentrations ranging from 25 to 150 mg kg^−1^ dry mass. The washed seeds were then spread in Petri dishes (5 cm in diameter) covered with moistened and contaminated cotton and allowed to germinate in the dark at 25–30°C. The garden cress seeds were watered daily with deionized water. Sprouted seeds were collected on day 6 and oven-dried overnight at 45°C before being ground.

### Germination index determination

The germination index (GI) of the garden cress seeds has been examined over 6 days of germination under different Cd and Pb concentrations (25–150 mg/kg), GI was measured according to the Salehzade et al. equation^24^: GI = Σ (n_i_ / D_i_). Where n_i_ is the number of germinated garden cress seeds at day ‘i’ and D_i_ is the day ‘i’

#### Methanol extracts of 6-day-garden cress sprouts (GCS)

Two grams of each treatment of dried GCS were added to a flask containing 80% methanol (20 mL) and continuously shaken (120 rpm) overnight at 25–30°C. Each methanol extract was filtered three times using Whatman-one filter paper^23^.

#### Determination of total phenolic content

The Folin–Ciocalteu method of Velioglu et al.^25^ was used to determine total phenolic content. A mixture containing each methanol extract (50 µL), distilled water (850 µL), and Folin–Ciocalteu reagent (100 µL) was incubated at 25–30°C for 5 min. A sodium carbonate solution (20%) was then added to the mixture, which was maintained for 30 min at 25–30°C. The absorbance of each mixture was recorded at 750 nm. A gallic acid equivalent (GAE) standard curve was used to calculate total phenolic content, which was expressed as mg GAE 100 g DW^−1^.

#### Determination of total flavonoid content

Methanol extract (250 µL), 5% NaNO_2_ solution (75 µL), and distilled water (1.25 mL) were incubated at 25–30°C for 5 min. Aluminum chloride solution (10%) and NaOH solution (1.0 M) were then added to the mixture, which was maintained for 10 min at 25–30 °C. The absorbance of each mixture was recorded at 510 nm^26^. A catechin equivalent (CE) standard curve was used to calculate total flavonoid content, which was expressed as mg CE 100 g DW^−1^.

### Assays of antioxidant activity

#### DPPH assay

Methanol extract (50 µL) was added to 950 µL of 0.1 mM of DPPH (1, 1-diphenyl-2-picrylhydrazyl) solution and incubated for 30 min at 25–30°C in the dark^27^. The absorbance of the mixture was recorded at 517 nm. The DPPH radical-scavenging activity percentage = (Control absorbance− sample absorbance/control absorbance) ×100.

#### ABTS assay

Methanol extract (10 µL) was added to 990 µL of ABTS (2,2-azino-bis (3-ethylbenzo-thiazoline-6-sulfonic acid) reagent and incubated for 1 min at 25–30°C^28^. The absorbance of the mixture was recorded at 734 nm. The ABTS radical-scavenging activity percentage was calculated using the formula given for the DPPH assay (above).

The IC_50_ value is the phenolic concentration required to scavenge 50% of either DPPH or ABTS free radicals.

#### PMC assay

A phosphor-molybdenum complex (PMC) antioxidant assay was conducted according to Prieto et al.^29^ to determine antioxidant activity. The methanol extract (50 µL) was incubated with 950 µL of 28 mM sodium phosphate, 4 mM ammonium molybdate, and 600 mM sulfuric acid for 45 min at 90°C. The mixture was then cooled before absorbance was read at 695 nm. The EC_50_ was considered the phenolic concentration equivalent to 0.5 OD at 695 nm.

#### Total antioxidant activity

Following the work of Abdel-Aty et al.^23^, total antioxidant activity was calculated as follows: total antioxidant activity = total phenolic content (mg GAE 100 g DW^−1^)/mg IC_50_ or mg EC_50_.

#### Preparation of crude enzyme extract

According to the method of Abdel-Aty et al.^23^, one gram of GCS was homogenized with 10 mL of extraction buffer (50 mM Tris–HCl, pH 7.0 containing 1 mM EDTA) using a glass mortar under cooled conditions. After centrifugation (12,000 *g* for 12 min at −4ºC), each supernatant (crude enzyme extract) was stored at −20ºC until further use.

#### Assays of antioxidant enzymes

Peroxidase (POD; EC 1.11.1.7) activity was estimated following the method described by Miranda et al.^30^. The assay was performed in 50 mM sodium acetate buffer (pH 5.5) in the presence of two substrates, 8 mM H_2_O_2_ and 40-mM guaiacol, as well as crude enzyme extract. The increase in the absorbance at 1.0 per min at 470 nm was considered as one unit. Catalase (CAT; EC 1.11.1.6) activity was estimated using the method described by Bergmeyer^31^. The assay was performed in 75 mM phosphate buffer (pH 7.0) in the presence of 25 mM H_2_O_2_ as a substrate as well as crude enzyme extract. The decrease in absorbance at 0.1 per min at 240 nm was considered as one unit. Glutathione-S-transferase (GST; EC 2.5.1.18) activity was estimated according to the method described by Habig et al.^32^ The assay was conducted in 0.1 M potassium phosphate buffer (pH 6.5) and 1.6 mM GSH, with 1 mM CDNB added to the crude enzyme extract. The increase in absorbance was read at 340 nm. The enzyme concentration that converted 1 µM CDNB per min was considered as one unit. Glutathione reductase (GR; EC 1.6.4.2) activity was estimated using the method described by Zanetti^33^. The reaction was based on the oxidation of NADPH in the presence of GSSG as a substrate. The reaction mixture included 50 mM potassium phosphate buffer (pH 7.0) containing 1 mM EDTA, GSSG, NADPH, and crude enzyme extract. The enzyme concentration that oxidized 1 µmol of NADPH per min was considered as one unit.

#### Determination of peroxidase by gel electrophoresis

To identify peroxidase isozyme variations between the control and metal treatments of GCS, native polyacrylamide gel electrophoresis (native-PAGE) was performed according to the method of Stegemann et al.^34^ with a slight modification. The substrate solution contained benzidine HCl (0.25 g), 4 mL of glacial acetic acid, and distilled water (added up to a total volume of 50 mL). After electrophoresis, the gel was placed into the substrate solution along with 10 drops of hydrogen peroxide. The gel was incubated at room temperature until bands appeared.

#### Determination of total heavy metals

Before metal determination, all samples were digested using nitric acid, an acceptable matrix for consistent recovery of metals that are compatible with the analytical method^35^. All heavy metal analyses were performed on an Agilent 5100 Inductively Coupled Plasma–Optical Emission Spectrometer with Synchronous Vertical Dual View. For each series of measurements, an intensity calibration curve was constructed that was composed of a blank and three or more standards from Merck (Germany). The accuracy and precision of the metal’s measurements were confirmed using external reference standards from Merck; standard reference materials for trace elements in water and quality control samples from the National Institute of Standards and Technology were used to confirm the instrument reading.

All experimental procedures were carried out in compliance with relevant guidelines.

### Statistical analysis

Data were analyzed using one-way ANOVA followed by Tukey’s post hoc test; these tests were conducted in GraphPad Prism version 5. Data are presented as means ± SD (n = 4) and differences were considered significant at *p* < 0.01.

## Results and discussion

### Total phenolic and flavonoid contents

Table 1 screens the total phenolic and flavonoid contents of GCS grown in media contaminated with Cd and Pb at various concentrations (25–150 mg kg^−1^). The total phenolic and total flavonoid contents of untreated GCS as a control (1600 ± 51 mg GAE 100 g^−1^ DW and 231±6.6 mg CE 100 g^−1^ DW, respectively) significantly increased (*p* < 0.01) and reached their highest levels at the highest doses of Cd (3196±54 mg GAE 100 g^−1^ and 590±10.2 mg CE 100 g^−1^ DW, respectively) and Pb (3960 ± 76 mg GAE 100 g^−1^ and 522 ± 10.5 mg CE 100 g^−1^ DW, respectively) by 2.0, 2.6 and 2.5, 2.3-fold, respectively. Similarly, over-expression of phenolics and flavonoids was detected at 2.48–2.50-fold and 1.5–2.0-fold, respectively, above the levels in controls in C4 weed subjected to aluminum stress^36^. Accumulation of antioxidant-phenolic compounds in tissues facilitates the ability of plants to tolerate and detoxify Cd and Pb stress via their metal chelating activity and/or their antioxidant activity^37^. The hydroxyl and carboxylic groups of the phenolic compounds assist with the binding of metals. In addition, flavonoids, as an important class of antioxidant-phenolic compounds, aid the detoxification of ROS free radicals that are induced by heavy metal stress. Rice, as a cereal plant, responds to cadmium stress by accumulating antioxidant-phenolic compounds^38^. An increase in phenolic compounds is correlated with increases in Cd and Pb concentrations, which suggests that *de novo* synthesis of soluble phenolic compounds and/or hydrolysis of conjugated phenolic compounds occurs under heavy metal stress^39^. In addition, the increase in the soluble phenolic compounds that are used in the lignin biosynthesis of cell walls to create physical barriers against the harmful action of heavy metals has also been reported^40^. From such observations, we can conclude that the total phenolic and flavonoid contents of GCS are highly sensitive markers for indicating Pb and Cd contamination in soils, even when concentrations are low.

**Table 1:** Total phenolic and flavonoid contents of GCS germinated under different concentrations of Cd and Pb (25 to 150 mg kg^−1^).

Metal mg kg^−1^	Total phenolic mg GAE 100 g^−1^ DW	Total flavonoid mg CE100 g^−1^ DW
Control	1600 ± 51^a^	231 ± 6.6^a^
Cd_25_	2405 ± 80^b^	342 ± 10.2^b^
Cd_50_	2567 ± 83^c^	410 ± 11.3^c^
Cd_75_	2867 ± 92^d^	485 ± 12.6^d^
Cd_100_	2980 ± 65^e^	531 ± 18.1^e^
Cd_150_	3196 ± 54^f^	590 ± 10.2^f^
Pb_25_	2400 ± 80^g^	360 ± 12.7^g^
Pb_50_	2587 ± 92^h^	395 ± 13.6^h^
Pb_75_	2752 ± 88^j^	426 ± 14.8^j^
Pb_100_	3066 ± 79^k^	473 ± 11.2^k^
Pb_150_	3960 ± 76^i^	522 ± 10.5^i^

GAE, gallic acid equivalent; CE, catechin equivalent; Cd, cadmium; Pb, lead. Data are presented as means ± SD (n = 4) and values in the same column with different superscripts were considered significant at *p* < 0.01.

### Antioxidant activities

Most of the recently identified phenolic compounds of GCS have potent antioxidant activity^23^. Therefore, the antioxidant activity of GCS germinated in media contaminated with Cd and Pb was evaluated by various antioxidant assays. In Table 2, IC_50_ values using DPPH and ABTS methods and EC_50_ values using the PMC method of untreated GCS (0.0098, 0.0065, and 0.007 mg GAE mL^−1^, respectively) gradually and significantly decreased (*p* < 0.01) and reached to their lowest values (0.0041, 0.0011 and 0.0021 mg GAE mL^−1^, respectively) at highest concentrations of Cd and Pb, respectively. Low IC_50_ and EC_50_ values reflect a high antioxidant activity. Additionally, the total antioxidant activity of GCS gradually and significantly increased relative to the control levels (*p* < 0.01) to reach maximum levels for Cd by 6.1-, 13.0-, and 5.8-fold and for Pb by 5.9-, 14.6-, and 8.2-fold at the highest concentrations of Cd and Pb (Table 3). This increase in antioxidant activity may be attributed to increases in the concentration of antioxidant-phenolic compounds that were associated with increasing Cd and Pb concentrations in growth media. ABTS and DPPH free radicals were tested in all antioxidation reactions of organic residues that were combined with ROS radicals^41,42^. Therefore, the antioxidant activities of GCS could be used as potential bioindicators for Cd and Pb toxicity. In previous studies, the antioxidant activity of rice was found to increase during Cd stress, which facilitated metal chelating and enhanced Cd tolerance^38^. Total antioxidant activity increased from 1.2- to 1.7-fold in *Malva parviflora* roots and leaves under different Cd concentration treatments^43^. Additionally, ABTS and DPPH free radical scavenging activity as well as PMC reduction activity gradually increased in C4 weed grown under aluminum treatments^36^. In contrast, chickpeas grown under different heavy metals treatments had an antioxidant activity that remained lower than that of the control, according to DPPH or ABTS assays, but that slightly increased as the concentration of accumulated metal increased^44^.

**Table 2:** The antioxidant activity of the phenolic content for GCS germinated under different concentrations of Cd and Pb (25 to 150 mg kg^−1^).

Metal mg kg^−1^	IC50 (mg GAE mL^−1^)		EC50(mg GAE mL^−1^)
	DPPH	ABTS	PMC
Control	0.0098 ± 2.0 × 10^−4a^	0.0065 ± 5.0 × 10^−5a^	0.0070 ± 6.0 × 10^−4a^
Cd_25_	0.0073 ± 1.4 × 10^−4b^	0.0048 ± 3.4 × 10^−5b^	0.0060 ± 5.3 × 10^−4b^
Cd_50_	0.0063 ± 1.3 × 10^−4c^	0.0036 ± 2.2 × 10^−5c^	0.0050 ± 5.0 × 10^−4c^
Cd_75_	0.0052 ± 1.1 × 10^−4d^	0.0025 ± 1.7 × 10^−5d^	0.0044 ± 4.2 × 10^−4d^
Cd_100_	0.0041 ± 1.2 × 10^−4e^	0.0015 ± 2.4 × 10^−5e^	0.0033 ± 4.2 × 10^−4e^
Cd_150_	0.0032 ± 1.1 × 10^−4f^	0.0010 ± 3.0 × 10^−5f^	0.0024 ± 4.1 × 10^−4f^
Pb_25_	0.0080 ± 1.8 × 10^−4 g^	0.0045 ± 3.1 × 10^−5 g^	0.0059 ± 5.1 × 10^−4b^
Pb_50_	0.0071 ± 2.1 × 10^−4b^	0.0032 ± 3.2 × 10^−5 h^	0.0050 ± 5.8 × 10^−4c^
Pb_75_	0.0062 ± 2.0 × 10^−4c^	0.0023 ± 2.7 × 10^−5j^	0.0041 ± 6.2 × 10^−4j^
Pb_100_	0.0051 ± 2.1 × 10^−4d^	0.0016 ± 2.8 × 10^−5 k^	0.0032 ± 5.8 × 10^−4e^
Pb_150_	0.0041 ± 2.2 × 10^−4e^	0.0011 ± 2.9 × 10^−5i^	0.0021 ± 6.0 × 10^−4 g^

*GAE* Gallic acid equivalent. Data are presented as means ± SD (n = 4) and values in the same column with different superscripts were considered significant at *p* < 0.01.

**Table 3:** Total antioxidant activity of GCS germinated under different concentrations of Cd and Pb (25 to 150 mg kg^−1^).

Metal mg kg^−1^	Total antioxidant activity (Total phenolic content of 100 g DW /IC50 or / EC50)		
	DPPH	ABTS	PMC
Control	163,265.3 ± 3200^a^	246,153.8 ± 2020^a^	228,571.4 ± 4571^a^
Cd_25_	307,397.3 ± 3143^b^	467,500.0 ± 4700^b^	374,000 ± 3680^b^
Cd_50_	407,460.3 ± 4140^c^	713,055.5 ± 5022^c^	513,400 ± 5101^c^
Cd_75_	551,346.2 ± 5012^d^	1,146,800 ± 9043^d^	651,591 ± 6012^d^
Cd_100_	726,829.3 ± 6940^e^	1,198,666 ± 9110^e^	903,030 ± 6210^e^
Cd_150_	998,750 ± 3033^f^	3,196,000 ± 9700^f^	1,331,667 ± 8312^f^
Pb_25_	300,000.0 ± 6022^b^	533,333.3 ± 5065^g^	406,779 ± 5275^g^
Pb _50_	364,366.2 ± 5940^g^	808,437.5 ± 5530^h^	517,400 ± 4956^c^
Pb_75_	443,871.0 ± 5031^h^	1,196,522 ± 7300^e^	671,220 ± 5809^d^
Pb_100_	601,176.5 ± 4540^j^	1,916,250 ± 7600^j^	958,125 ± 7632^h^
Pb_150_	965,854 ± 6000^f^	3,600,000 ± 8456^k^	1,885,714 ± 7737^j^

Data are presented as means ± SD (n = 4) and values in the same column with different superscripts were considered significant at *p* < 0.01.

### Antioxidant enzymes

**Figure 1:**
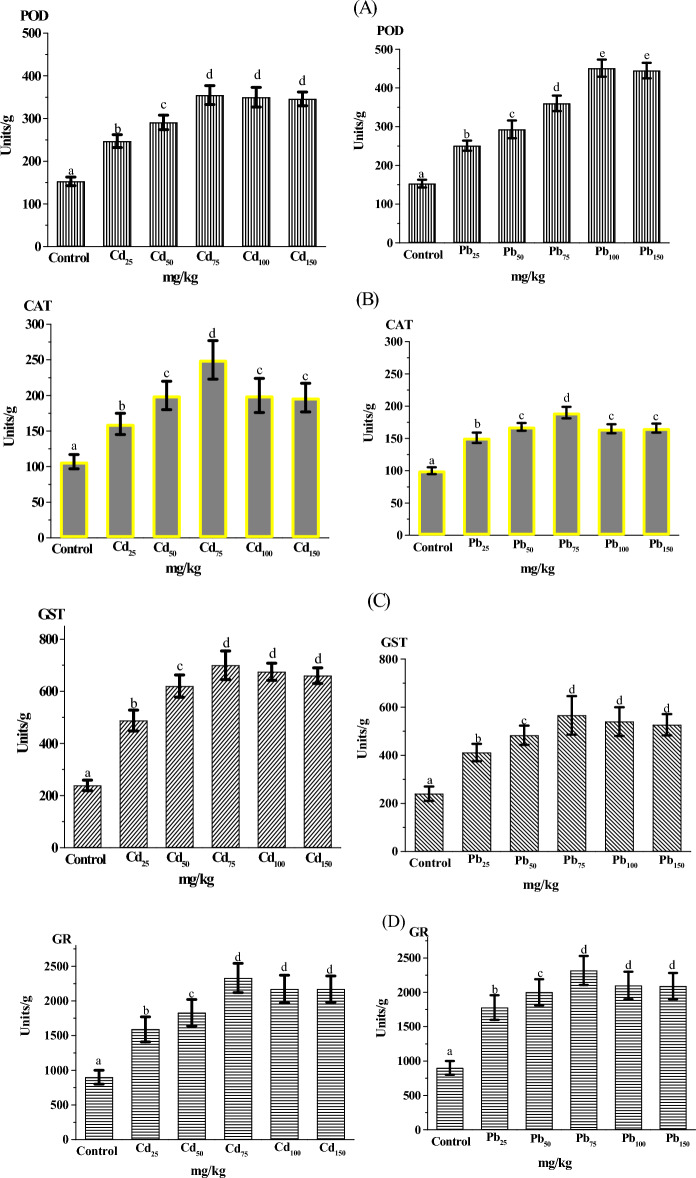
Effect of Cd and Pb at different concentrations (25-150 mg kg^−1^) on the activities of the antioxidant enzymes: (**A**) POD, (**B**) CAT, (**C**) GST, and (**D**) GR of GCS. Data are presented as means ± SD (n = 4) and differences were considered significant at *p* < 0.01.

Cd and Pb can cause the overproduction of ROS free radicals and induce oxidative damage to plant tissues. Maintaining the balance between ROS free radicals and activation of the antioxidative system under heavy metal stress is a critical protective mechanism in plants that diminishes oxidative damage in polluted tissues^15^. Therefore, in the present work, the potential role of the antioxidant enzymes (POD, CAT, GR, and GST) in response to oxidative stress in GCS under Cd and Pb treatments was investigated; the results are presented in Fig. 1. POD activity gradually increased (*p* < 0.01) with increasing of Cd and Pb concentrations till reached highest activity (355 and 451 U g^−1^, respectively) compared to the control (153 U g^1^) (Fig. 1A). The results observed a strong correlation between the phenolic content and POD activity of GCS germinated under Cd and Pb treatments. POD is involved in removing H_2_O_2_ as a harmful ROS-free radical by oxidation of phenolic compounds^45–48^. Similarly, increasing Hg accumulation in garden cress shoots was correlated with an increase in POD activity and changes in total carotenoid content^19^. Furthermore, POD participates in the polymerization of phenolic compounds for lignin synthesis to build a barrier that protects against toxic metal ions^49^.

In the antioxidant defense system, CAT also stopped the hyper-accumulation of H_2_O_2_ by converting it to water and oxygen. Here, compared with control CAT activity (100 U g^−1^), an increase in Cd and Pb treatment concentrations caused a significant increase (*p* < 0.01) in CAT activity, which peaked (250 and 190 U g^−1^, respectively) at 75 mg metal kg^−1^ (Fig. 1B). Similarly, CAT and POD activities were significantly enhanced in vetiver grass, *Populus nigra*, *Cajanus cajan*^50–52^, and *Malva parviflora*^43^ grown in Cd- and/or Pb-contaminated soils. This considerable increase in CAT activity could be induced by excessive production of H_2_O_2_ under 75 mg Cd and Pb kg^−1^. However, the CAT activity was reduced under higher concentrations of Cd and Pb (100 and 150 mg kg^−1^), which may be due to severe oxidative damage occurring under much higher Cd and Pb levels. The severity of the Cd/Pb stress may suppress enzyme synthesis or alter the enzyme's assembly^53^. Interestingly, this decline in CAT activity was compensated by the increase in POD activity to remove H_2_O_2_. Many previous investigations on some plant species have revealed that variations in antioxidant enzyme activities were related to the severity of Cd/Pb stress^53,54^.

The important GSH-utilizing enzymes, GST and GR, were also evaluated in the current study. These enzymes largely participate in the efficient metabolism of ROS free radicals and their products^55^. Hence, they tightly control heavy metal-induced oxidative stress in plants. The activities of GST and GR enzymes significantly increased relative to control levels (239 and 900 U g^−1^, respectively) in all Cd and Pb treatments, and the activities reached their peak at 75 metal mg kg^−1^ treatments (Cd: 700 and 566 U g^−1^, respectively; Pb: 2,330 and 2,318 U g^−1^, respectively) (Fig. 1C and D). An increase in GST activity was previously reported in pumpkin, rice, and *Cicer arietinum* in response to either Cd or Pb stress^17,56,57^. Our results revealed a strong correlation between the phenolic compound levels and GST activity of GCS under Cd and Pb treatments, suggesting that GST not only removes toxic ROS radicals but also transports the phenolic compound chelating-metal complexes to the vacuoles^58^. In a previous study, GR activity increased in *Brassica napus* leaves under Cd stress^59^. In contrast, GR activity decreased in *Ceratophyllum demersum* and mung bean seedlings in response to Cd stress^60,61^. Such variations in GR activity and responses may have been due to differences in plant genotypes^58^. In the results described above, there was a direct relationship between Cd and Pb concentrations and POD, CAT, GST, and GR activities, which indicates that these four enzymes of the antioxidant defense system could be used as bio-indicators for Cd and Pb stress. In addition, the GCS can cope with the oxidative damage induced by Pb and Cd.

### Electrophoretic pattern of peroxidase activity

**Figure 2:**
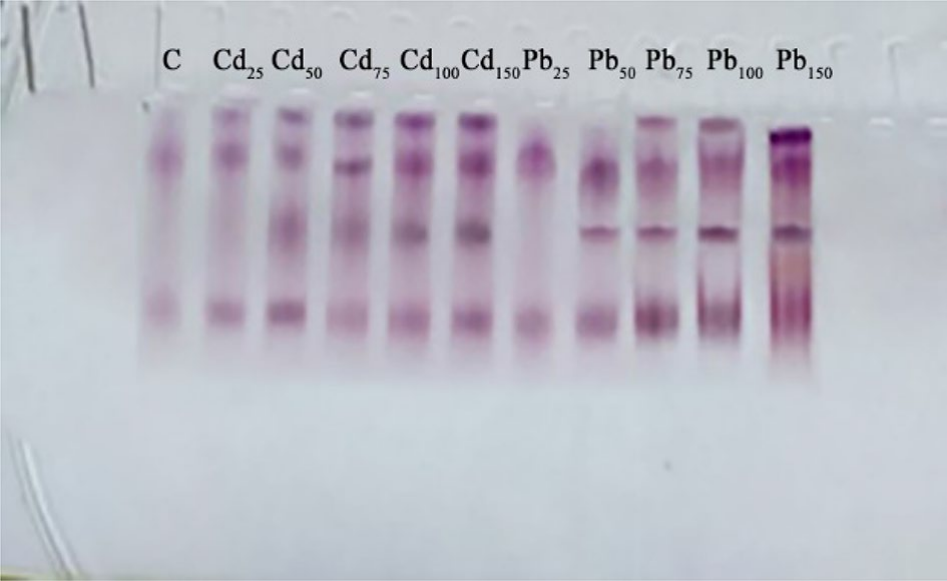
Electrophoretic patterns of the POD of GCS germinated under different concentrations of Cd and Pb (25 to 150 mg kg^−1^) compared to untreated control (C).

Fig. 2 shows the electrophoretic patterns of peroxidase in GCS germinated under Cd and Pb treatments (25–150 mg kg^−1^) compared with untreated controls. Two peroxidase isozymes were detected in untreated control and the intensity of these bands increased gradually with increasing Cd and Pb concentrations; moreover, two new peroxidase isozymes appeared. This observation explains why POD activity gradually increased in all Cd and Pb treatments to cope with ROS that caused stress-related damage to plant tissues.

### Bioaccumulation of Cd and Pb in GCS

**Figure 3:**
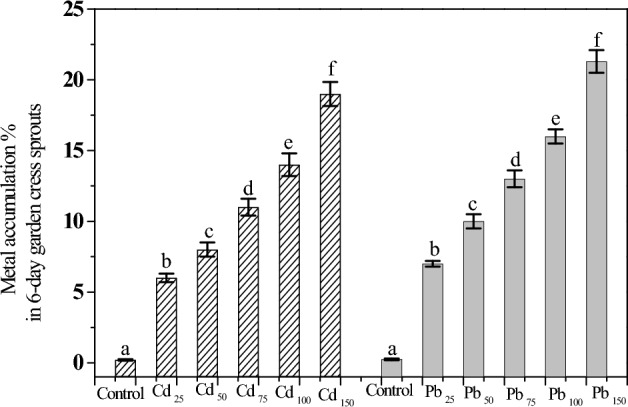
Cd and Pb accumulation percentage in tissues of GCS germinated under different concentrations of Cd and Pb (25 to 150 mg kg^−1^). Data are presented as means ± SD (n = 4) and differences were considered significant at *p* < 0.01.

The contents of Cd and Pb absorbed by GCS showed a significant increase (*p* < 0.01) with increasing Cd and Pb at 25, 50, 75, 100, and 150 mg kg^−1^ in the order of 6.0, 8.0, 11.0, 14.0, 19.0 and 7.0, 10.0, 13.5, 16.0, 21.3%, respectively (Fig. 3). Thus, GCS seem to have the ability to take up Cd and Pb from contaminated media as phytoremediator in a short germination period (6 days). A previous study reported that garden cress plants could absorb (through their shoots and roots) 48% of Pb from soil contaminated with a 300-mg kg^−1^ concentration after a 30-day culture^20^. Another study found that garden cress reduced 100-mg kg^−1^ Hg by 33% in contaminated growth media after six repeated phytoextraction processes^19^. Additionally, chickpea seedlings produced maximum uptake of Pb (~3.8%) from contaminated soil at a Pb concentration of 250 mg kg^−1^^44^.

### Visual changes of GCS and germination index

**Figure 4:**
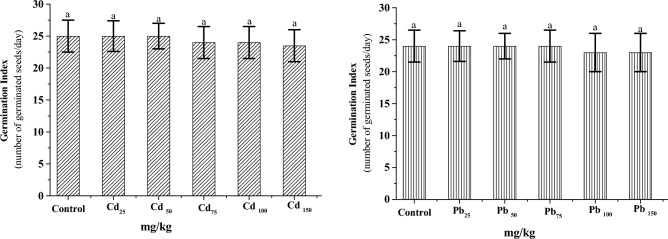
Germination index of GCS germinated under different concentrations of Cd and Pb (25 to 150 mg kg^−1^). Data are presented as means ± SD (n = 4) and differences were considered significant at *p* < 0.01.

During six days of germination, the GCS did not exhibit any noticeable visual changes when subjected to different concentrations of Cd and Pb, even under substantially higher concentrations of both. In addition, the germination index (GI) of the GCS was conducted throughout 6 days of germination under different Cd and Pb concentrations (25–150 mg/kg), as seen in Fig. 4, and the results showed that the GI was unaffected by these heavy metals even at their higher doses. One could conclude that the potent antioxidant defense system of GCS could easily counter the damaging effects of Cd and Pb toxicity. Some plants exposed to toxic levels of Cd and Pb decrease germination and interfere with seedling physiological processes^62^ such as cowpea, soybean, lettuce, and sugar beet^8^. While parsley seedlings that grew under considerably higher Cd concentrations didn't show any signs of visual changes^63^.

### Bio-indicators collective data

All of the tested parameters that were measured in GCS germinated under the lowest Cd and Pb concentrations (25 mg kg^−1^) observed fold increase values of 1.5–2.2-fold (Table 4). At the highest Cd and Pb concentrations (150 mg kg^−1^), the fold-increase values of these parameters were slightly higher (1.7–2.9-fold), but the expected substantial increase did not occur, except for total antioxidant activity (5.8–14.6-fold) (Table 4). These observations suggest that all the tested parameters of GCS responded strongly to lower concentrations of Cd and Pb and the antioxidant activity of phenolic compounds of GCS can be considered a potent bioindicator for monitoring Cd and Pb at both low and high concentrations. It is important to conduct these experiments outdoors in varied soils under different environmental conditions in order to study more realistic changes in the GCS-antioxidant system and the accumulation and distribution of these heavy metals.

**Table 4:** Collective data on the fold-increase among all the tested parameters at the lowest and highest concentrations of Cd and Pb in comparison to their controls.

Metal mg kg^−1^	Total phenolic content	Total flavonoid content	Fold increases in total antioxidant activity			Antioxidant enzymes			
			DPPH	ABTS	PMC	POD	CAT	GST	GR
Cd_25_	1.5^a^	1.5^a^	2.0^a^	2.0^a^	1.6^a^	1.6^a^	1.5^a^	2.0^a^	1.8^a^
Cd_150_	2.0^b^	2.6^b^	6.1^b^	13.0^b^	5.8^b^	2.2^b^	2.3^b^	2.8^a^	2.5^b^
Pb_25_	1.5^a^	1.6^a^	1.8^a^	2.2^a^	1.8^a^	1.7^a^	1.5^a^	1.7^a^	2.0^a^
Pb_150_	2.5^b^	2.3^b^	5.9^b^	14.6^b^	8.2^b^	2.9^b^	1.7^b^	2.2^b^	2.3^b^

Data are presented as means ± SD (n = 4) and values in the same column with different superscripts were considered significant at *p* < 0.01.

## Conclusion

In the present work, GCS was germinated with different concentrations of Cd and Pb (25–150 mg kg^−1^) under *in vitro* conditions. The phenolic and flavonoid levels and antioxidant capacity were enhanced several folds and correlated with the Cd and Pb concentrations. The activities of antioxidant enzymes (POD, CAT, GR, and GST) were strongly increased under Cd and Pb stress. In addition, new peroxidase isozymes appeared in the electrophoretic profile. Further, considerable amounts of Cd and Pb were absorbed by the tissues of GCS. Moreover, all the investigated parameters of GCS responded considerably to lower levels of Cd and Pb, and the GCS-antioxidant activity was a potent bioindicator to monitor Cd and Pb at low and high levels. Based on these findings, we conclude that metal stress alters the biochemical parameters of GCS, which could be used as bio-monitors for Cd and Pb soil contaminants within short germination periods.

## Data Availability

The datasets generated during and/or analyzed during the current study are available from the corresponding author upon reasonable request.
